# From gut dysbiosis to eubiosis: understanding microbiome recovery as an ecological process

**DOI:** 10.1080/19490976.2026.2649448

**Published:** 2026-04-22

**Authors:** Megan S. Kennedy, Eugene B. Chang

**Affiliations:** a Medical Scientist Training Program, Pritzker School of Medicine, The University of Chicago, Chicago, IL, USA; b Department of Medicine, The University of Chicago, Chicago, IL, USA

**Keywords:** Microbiome recovery, antibiotics, diet, inflammation, perturbation, disturbance ecology, stability, resilience, prehabilitation, community assembly, secondary succession

## Abstract

Predicting and promoting gut microbiome recovery following perturbations such as antibiotic treatment, dietary shifts, or inflammation remain major challenges in microbiome science and clinical practice. In this review, we explore recent advances in microbiome restitution by framing recovery as a dynamic ecological process shaped by complex interactions between microbial taxa, host physiology and environmental conditions. We review current evidence addressing four key questions that outline the salient ecology of perturbation and recovery: which microbial taxa are present in the microbiota and which taxonomic or functional qualities might increase susceptibility to perturbation; what is the nature of the perturbation, including the type and probable targets of the perturbation, as well as the indirect ecological and environmental consequences of that perturbation; what is the time course of perturbation and recovery, exploring prehabilitation strategies and successional trajectories as a staged recovery framework; and where does perturbation and recovery unfold in the gut, with attention to both regional and microscale spatial patterns. Highlighting recent advances from multi-omics approaches and longitudinal studies, we demonstrate how each of these factors and their interactions critically shape both robustness to disturbance and the trajectory of recovery. We advocate for multimodal, context-specific interventions that harness ecological principles to drive regrowth and community assembly, including diet, targeted microbial transplantation and modulation of the abiotic gut environment. Ultimately, resolving the challenge of microbiome restitution will require personalized strategies informed by ecological understanding and longitudinal functional monitoring. This paradigm provides a foundation for future translational advances to promote eubiosis and improve patient outcomes in microbiome-related diseases.

## Introduction

The gut microbiota is increasingly appreciated as an essential organ of the human body, playing an orchestrating role in physiological and developmental processes spanning the gastrointestinal (GI) tract^1^, metabolism^2^, the immune system^3-6^, the nervous system^7^^,^^8^, and more^9^. However, this evolutionarily complex organ system exists in delicate homeostatic balance with the host and external factors, and perturbations of many kinds can induce compositional and functional alterations broadly referred to as “dysbiosis.” A dysbiotic microbiota may lose its ability to perform essential metabolic or immunomodulatory functions^2^^,^^10^^,^^11^ or may even take on new and harmful functionality^12^^,^^13^ or alternative states of stability and resilience that are difficult to reverse.

Antibiotics, while essential pillars in the management of infectious disease, can inflict severe collateral damage to the microbiota, with consequences ranging from inconvenient (e.g., diarrhea and gas^14^) to life-threatening in some cases of opportunistic intestinal^15-18^ or bloodstream infection^19^. Persistent changes in microbial community structure following antibiotics have also been linked to broader physiologic changes in host metabolism^20-22^, GI motility^23^^,^^24^, allergy^25-27^, and inflammation^28^^,^^29^. Other endogenous perturbations, such as inflammation^30^^,^^31^ or hormonal change^32^, or exogenous perturbations, such as a shift in diet^33^, medications^34^, pre-endoscopic bowel prep regimens,^35^ GI surgery^36^, or infectious diarrheal disease,^37^ can similarly wreak havoc on the gut ecosystem.

As the impact of these disturbances on the microbiota has become better characterized and the associations between dysbiosis and pathologic outcomes better documented, identifying interventions to address dysbiosis and promote microbiome recovery has emerged as a priority for researchers in the field. Progress, however, has been slow, leaving many questions broadly unanswered. Why do some people have a microbiota that recovers from perturbation in days, while others experience complications that last for months or even years?^38-40^ Which interventions are most effective for promoting recovery or preventing dysbiosis in the first place, and when should they be optimally introduced?

**Figure 1.**
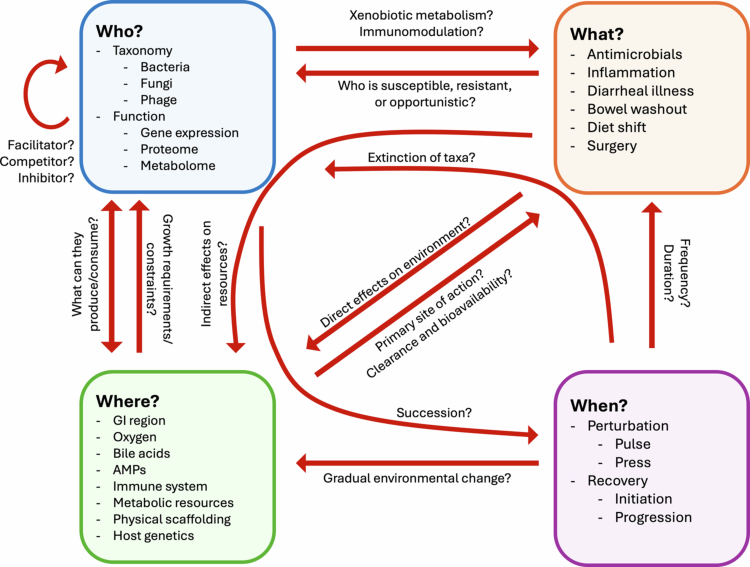
**Who, what, where, and when.** We present a heuristic series of questions to center inquiry on the dynamic ecology of microbiome recovery. “Who” reflects the microbial agents, as well as their functional output, that are represented before and after a perturbation in the gut ecosystem. “What” represents the characteristics of the perturbation itself. “Where” reflects the “abiotic” or non-microbial characteristics of the ecosystem affected by the perturbation. “When” refers to the time course of the perturbation itself, as well as the time course and progression of the subsequent recovery dynamics. Arrows reflect potential constraints between elements of this framework. Curved arrows reflect constraints that may act indirectly across multiple features of the framework.

Here, we argue that to better understand, and therefore drive, microbiome recovery, we must understand recovery not as a state but as an ecological process. The decimation of a forest fire cannot simply be undone: rather than seeking to reverse the dysbiosis that results from perturbation of the microbiota, we must learn to cultivate the regrowth of a vibrant and diverse microbial community. In this review, we propose several critical questions—who, what, where, and when—that act to characterize the salient ecology of the microbiota before, during, and after a perturbation and how researchers have answered them across clinical settings (Figure 1). We hope to demonstrate that by asking the right questions, we can arrive at essential insight into contextually unique recovery processes, paving the way for the development of safe, effective, and perhaps even personalized interventions to promote microbiome restitution.

## Who: which microbes are present in the community?

**Figure 2.**
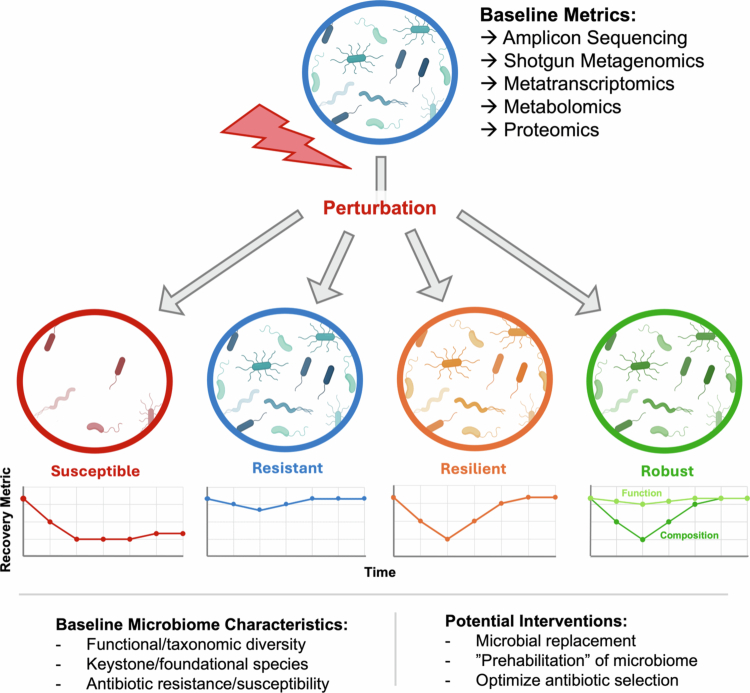
**Baseline microbiota characteristics impact recovery.** Schematic representation of communities that are susceptible to perturbation (undergo dramatic community shift after perturbation, without recovery), resistant to perturbation (experience minimal change in community structure and recover rapidly), resilient (undergo substantial change in community structure in response to perturbation but recover to baseline status), or robust (undergo substantial change in community structure but maintain salient functional output). Baseline measures of eubiosis can be assessed using taxonomic methods (16S rRNA amplicon sequencing, shotgun metagenomics) or more functionally informed metrics (metatranscriptomics, metabolomics, proteomics). Differences in characteristics such as the baseline level of functional or taxonomic diversity in the community, the presence or absence of foundational species that play an essential role in community reassembly, and the baseline presence of taxa that are more or less resistant to particular antibiotics or types of perturbations, may all impact recovery. Intervention strategies may focus on replacing taxa that are extinguished after perturbation, utilizing “prehabilitation” programs to optimize taxonomic or functional characteristics of the microbiota prior to planned perturbation, or efforts to select antibiotics more likely to spare microbial taxa dominant in that individual's gut community (within the limits of clinical efficacy).

First and foremost, to understand microbiome recovery, we must ask what the microbial community looked like both before the perturbation, in a state of relative health, and afterwards (Figure 2). A sufficient characterization of “who” is present in the microbiome entails determining not only which microbial taxa are present in the community, typically via 16S rRNA amplicon sequencing^41^ or short- and/or long-read metagenomic sequencing^42^, but also their functional potential and output, quantifiable using metagenomics, metatranscriptomics^43^, metabolomics^44^, or proteomic analyses^45^. Probing these datasets to quantify the abundance and diversity of antibiotic resistance genes (ARGs)^46^ and virulence factors^47^ may be particularly insightful for understanding microbiome recovery after antibiotic treatment and the emergence of opportunistic pathogens. And, although we focus primarily on bacteria in this Review, evaluation of the fungal mycobiome^48^ or virome^49^ may provide deeper insight into the full cast of ecological agents at play.

Given the extensive functional redundancy of the microbiota^50^^,^^51^ and evidence that the rates of taxonomic and functional recovery are at least somewhat decoupled after perturbation^52-54^, a growing number of researchers advocate for the prioritization of functional over taxonomic metrics of microbiome health^55^^,^^56^. However, as taxonomic shifts still fundamentally underlie functional potential,^57^^,^^58^ we caution against wholly dismissing taxonomic data, particularly in perturbation contexts that might entail taxonomic extinctions.

Collectively, this body of data provides a baseline for what a “healthy” or “recovered” microbiota should look like, taxonomically and functionally, serving as a useful barometer for both how severely a given perturbation has impacted the microbiome and how effectively a given intervention has promoted recovery. The impact of a perturbation on the microbiome can be described with specific terminology: we define a community that is “resistant” to perturbation as one that can withstand change in spite of perturbation. A community that is “robust” to perturbation maintains desirable function in spite of ecosystem change. A community that is “resilient” to perturbation can undergo an ecosystem shift of great magnitude or duration while still spontaneously returning to its initial state.

We note that for many individuals in a state of persistent dysbiosis, such as those with chronic inflammation^59^ or who have longstanding obesity,^60^ identifying a true “initial” state may be impractical, and the goal of recovery would certainly not be a return to a stable form of dysbiosis. Moreover, most clinical cases of microbiome perturbation (e.g., infectious illness and subsequent antibiotic treatment, emergency surgery, new-onset inflammation) are unplanned, creating logistical barriers to the collection of pre-disturbance data. In these cases, comparison to population-level data from healthy individuals rather than an individual’s own pre-disturbance state can establish a goal for eubiosis, and intervention can be directed towards engineering a new and more favorable alternative stable state.

The comparison of post-disturbance community characteristics with a goal recovery state allows us to determine whether desirable taxa or functions have been wholly extinguished from the community, for instance, after severe perturbations such as prolonged dietary fiber deficiency^61^ or an extended course of antibiotics.^62^^,^^63^ This, in turn, can help to guide decisions about whether endogenous regrowth remains a feasible option or whether exogenous microbial replacement might be necessary to restore extinct taxa or functions.

To date, most research on microbial replacement strategies has examined probiotics, synbiotics, and fecal microbiota transplant (FMT). Traditional probiotics are supplements containing live bacterial taxa typically derived from fermented foods^64^. Synbiotics pair probiotics with a “prebiotic” or a metabolic substrate intended to selectively promote the growth of the probiotic^65^. FMT entails bulk transplant of fecal material, including all bacterial,^66^ viral,^67^ and fungal^68^ components, from a “healthy” donor into the recipient. Although each of these methods has demonstrated promise in certain clinical contexts^69-71^, they suffer from variable efficacy in others^72-77^. This inconsistent track record may arise from a failure to explore the broader ecological aspects of microbiome recovery: which microbial taxa are being introduced, and which already exist in the recipient microbiota?^78-80^ Which regions of the GI tract will they colonize?^81^ When is the optimal timing to administer microbial replacement, and is one administration sufficient?^82^ What is the “abiotic” environment of the gut like, and can we leverage that information to promote better engraftment of transplanted microbes, For instance by employing concurrent immunomodulatory^83^ or dietary interventions?^84^^,^^85^ The development of “next-generation probiotics,” which expand the range of live bacterial supplements to include host-derived taxa and strains engineered to perform specific functions,^64^ may be better suited to answer these questions in a precise and tailored manner.

We might also exploit knowledge of which taxa remain or expand after a perturbation to develop subtractive methods of microbiome manipulation for the removal of opportunistic pathobionts. Organisms such as *Clostridioides difficile,*^86^ extended-spectrum beta-lactamase-resistant *Enterobacteriaceae,*^87^^,^^88^ *Klebsiella pneumoniae,*^89^ *Candida albicans,*^90^ or *Bacteroides fragilis*^91^, for example, are all indigenous members of the human gut microbiota that can bloom after antibiotic treatment or in inflammatory contexts, directly contributing to poor outcomes for the host. To control or remove such taxa, some researchers are exploring the use of precisely engineered bacteriophages, which are viruses that prey on bacteria.^92^ In one mouse study from Federici et al., researchers developed a cocktail of five lytic phages that was able to specifically target and eliminate inflammation-associated strains of *K. pneumoniae* without affecting benign commensal strains of the same species.^93^ Another phage cocktail called EcoActive is now in clinical trials (NCT03808103 on clinicaltrials.gov) after demonstrating the ability to specifically lyse colitis-associated adherent-invasive *E. coli* strains in mice.^94^

Characterization of the pre- and post-perturbation microbial community can also provide insight into which individuals or classes of individuals may be more susceptible to perturbation. For instance, overall microbiome robustness to antibiotic treatment depends critically on the abundances of taxa that are susceptible or resistant to that particular antibiotic^95-97^. High taxonomic diversity has long been used as a heuristic for microbiome health^50^^,^^98^^,^^99^, and several studies have shown that high initial diversity promotes robustness to perturbation,^100^^,^^101^ with improved defense against the emergence of opportunistic pathogens or antibiotic-resistant strains^102^^,^^103^. This effect may derive from multiple mechanisms—functional redundancy, or the capacity of many different taxa to perform the same functions, generally increases with taxonomic diversity^104^, buffering the functional output of an ecosystem against the loss of specific taxa^105^. A more diverse microbiota, fulfilling a broader array of functions, also leaves fewer unoccupied ecological niches for opportunistic pathogens to exploit. Thus, diversity, and especially functional redundancy, can promote the competitive exclusion of invading taxa or opportunistic pathogens.^106-108^ Other researchers argue that diversity *per se* is less important for robustness and stability than the specific interaction structure of microbial taxa^109^. Classic ecological theory posits that a more diverse system is less likely to be stable owing to competitive exclusion effects^110^^,^^111^. The robustness of diverse communities might instead derive from factors such as spatial^112^^,^^113^ and temporal^114^ niche partitioning, intransitive competitive interactions (i.e., rock-paper-scissor dynamics)^115-117^, or density-dependent predation by phage in a “kill-the-winner” structure that prevents any one strain from becoming overly dominant.^118^

As precision medicine transitions from aspiration to clinical reality,^119^ we can imagine a future in which ongoing, point-of-care evaluation of the microbiota is available and in which regular, personalized characterization of the “healthy” microbiome is not only feasible but also considered integral to a comprehensive annual health screening.

## What: what is the nature of the perturbation?

Fundamental to predicting the outcome of a perturbation is a deep understanding of the perturbation itself. Perturbation changes the selective pressures of the gut ecosystem, restructuring the niche space as conditions change and resulting in competitive advantages for certain microbial populations and disadvantages for others. Thus, for any perturbation, we must ask *what* the perturbation is, how it affects the “abiotic” conditions of the ecosystem (e.g., metabolic resource availability, pH, oxygen levels, GI motility, inflammatory milieu), and which populations we expect to bloom or decline under those conditions (Table 1). Combined with information about the pre-perturbation microbiota, this allows us to predict how we expect community structure to change after disturbance, both directly as a consequence of the perturbation and indirectly via rippling loss of connections in the microbial interaction network. This, in turn, lays essential groundwork toward predicting the trajectory of microbiome recovery by establishing which taxa are most likely be available for re-seeding, and whether or how altered ecosystem conditions may promote or prevent the reemergence of certain taxa.

**Table 1.** Summary table of different perturbation types.

Perturbation	Effects on microbiota and gut ecosystem	Recovery timelines	Potential intervention strategies
**Antibiotics**			
Broad-spectrum vs narrow spectrum, bacteriostatic vs bactericidal, *etc.*	- Reduced diversity, extinction of taxa^120^ - Open niche space^106^ - Loss of foundational taxa^246^ - Emergence of opportunists (pathogens, fungi)^86^^,^^90^ - Emergence of antibiotic resistance^257^^,^^258^	- Weeks to years^38-40^ - Depends on drug, dose, and duration of treatment^120^ - Extinctions require intervention^259^	- Fecal microbiota transplant^69^ - Dietary intervention (esp. fiber)^101^^,^^176^^,^^177^
**Diet**			
E.g., low-fiber, high fat	- Reduced diversity, extinction of taxa^33^^,^^61^ - Reduced SCFA production^260^ - Bacterial digestion/invasion of epithelial mucus barrier^131^ - Shift in bile acid pool^261^, emergence of bile-acid metabolizers^132^	- Days to weeks^61^ - Extinction require intervention^61^	- Dietary intervention^262^ - Microbial transplant with fiber supplementation^263^ - Postbiotic resource supplementation^149^
**GI inflammation**			
Ulcerative colitis, Crohn's disease	- Mucosal barrier disruption^139^ - Increased epithelial permeability, luminal oxygen^140^^,^^141^ - Immune infiltration of GI tissues^264^ - Emergence of pathogens/pathobionts with immune-escape^143^^,^^145^ - Emergence of metabolically autonomous taxa^142^ - Less stable microbiome configurations^265^	- Relapsing and remitting on scale of months to years^266^	- Immunomodulatory medications^267^^,^^268^ - Microbiome transplant^269^ - Dietary intervention^270^ - Mucosal barrier protection^199^ - Management of GI oxygen levels^209^ - Antibiotic treatment (pouchitis)^271^ - Phage therapy^93^^,^^94^
**Invasive procedures**			
E.g., bowel preparation, colonoscopy, major abdominal or colorectal surgery	- Mechanical flushing of taxa (bowel prep)^137^ - Removal of colonic niche (colorectal resection) - Shift in regional microbial colonization (pouchitis)^216^	- Weeks to months^272^	- Dietary pre- or peri-habilitation^239^^,^^240^ - Regional microbiota transplant^81^

Antibiotics again provide a useful example. Different classes of broad- or narrow-spectrum antibiotics explicitly target bacteria with certain characteristics (e.g., gram-positive/negative, aerobic/anaerobic, etc.) and can be either bactericidal (kill bacteria) or bacteriostatic (inhibit bacterial growth/reproduction). Correspondingly, different antibiotic regimens can have predictable impacts on microbiome community structure (reviewed extensively in Fishbein et al.^120^), often resulting in competitive advantages and overrepresentation of bacterial taxa with antibiotic resistance genes^63^^,^^121^ or fungi that are not directly impacted by antibacterial drugs and are released from bacterial competition.^122-125^ Antibiotic-induced changes in community structure can also modulate the abiotic conditions of the gut in ways that facilitate opportunistic infection: one mouse study from Rivera-Chavez et al. showed that antibiotic treatment depletes butyrate-producing *Clostridia* taxa.^126^ Colonocytes rely on butyrate as a primary energy source, oxidizing butyrate into CO_2_ and removing oxygen from the colon.^127^ Therefore, in antibiotic-treated mice without butyrate-producing *Clostridia*, the colon became more aerobic, allowing for the expansion of the facultatively aerobic opportunistic pathogen *Salmonella enterica* serovar *Typhimurium*^126^. Similarly, human data after antibiotic treatment document the expansion of facultative anaerobes,^95^^,^^128^^,^^129^ although explicit quantification of oxygen levels and validation of the specific mechanism of dysbiosis are currently lacking.

Researchers have documented predictable outcomes on microbiota composition for other types of perturbations as well, with implications for recovery. For example, in mice colonized with a human gut-derived microbial consortium, dietary changes that remove or minimize microbiota-accessible carbohydrates (MACs) favor the growth of bacteria such as *Akkermansia mucinophila* or certain *Bacteroides* genera that can metabolize host-derived mucosal glycans in lieu of dietary fiber^130^ at the cost of taxa such as *Eubacterium rectale*, which rely on dietary MACs.^131^ Switching mice to diets high in saturated fat causes increased taurine conjugation of host-derived bile acids, increasing the availability of organic sulfur and thereby promoting the growth of pro-inflammatory, sulfite-reducing pathobionts such as *Bilophila wadsworthia*^132^. Viral^133^ and fungal^134^^,^^135^ communities are likewise responsive to dietary changes. Clinical bowel washout regimens induce generalized loss of diversity as microbial taxa are flushed from the gut, with distinct microbiome changes across luminal and mucosal populations, possibly suggesting an advantage for populations with a stronger capacity to adhere to the mucosal surface.^136-138^

Inflammation of the distal GI tract induces a suite of physiologic changes, including disruption of the mucosal barrier^139^, increased epithelial permeability^140^, and increased oxygen levels^141^, resulting in a state that is stressful to many bacterial taxa. Among these, taxa that are metabolically “independent”, which can autonomously produce all the enzymes needed for the biosynthesis of essential organic compounds without reliance on the host or other bacteria, tend to be overrepresented.^142^ Taxa such as *B. fragilis* with potential mechanisms of immune escape, such as a variable polysaccharide capsule, have been found to bloom under inflammatory conditions,^143^^,^^144^ as do some opportunistic pathogens with latent virulence factors, such as *Salmonella enterica* or *C. albicans*, which in fact rely on active inflammation in order to invade host tissues^145^^,^^146^. Microbiome recovery in these cases may require timely microbiota-directed interventions concurrently with anti-inflammatory interventions such as corticosteroids or anti-TNF agents, as are commonly used in the treatment of IBD.^147^

**Figure 3.**
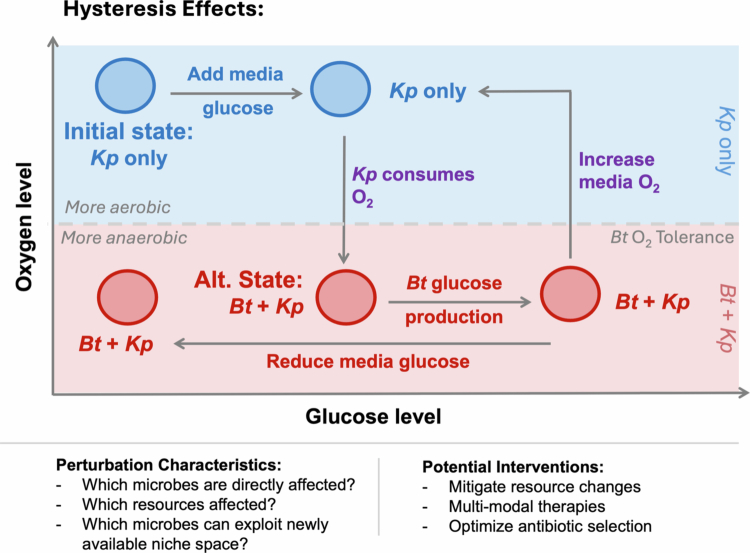
**Perturbation type affects optimal recovery strategies.** Different types of perturbations may exert different effects both directly on microbial taxa in the community, as well as on the resource environment of the gut. Hysteresis effects may occur when the microbiome profile depends not only on the conditions at the time of sampling but also on the history of those conditions. In this schematic example derived from Khazaei et al.,^273^ a community that begins with only the facultative anaerobe *Klebsiella pneumoniae* (*K. pneumoniae*) becomes anaerobic after increasing media glucose levels, as *K. pneumoniae* consumes glucose and oxygen via aerobic respiration. As the oxygen level decreases, the obligate anaerobe *Bacteroides thetaiotaomicron* (*B. thetaiotaomicron*) is able to grow, in turn releasing additional glucose through the breakdown of dextran in the media. Even when exogenous media glucose levels are reduced, the community is not able to return to its initial state because *B. thetaiotaomicron* is producing enough glucose for *K. pneumoniae* to maintain the anaerobic state. Thus, the only way to return to the initial state is by increasing the oxygen level. By evaluating which microbes are directly and indirectly affected by the shifting of the niche space after perturbation, we can identify strategies designed to minimize direct effects, ameliorate indirect effects, promote conditions that allow for recovery, and strategically combine microbiome- and GI-environment-directed therapies to best promote healing.

Notably, it may not be sufficient to simply stop or reverse the perturbation itself in order to stimulate recovery. An exquisite, if simplified, example of this phenomenon comes from Khazaei et al^148^ (Figure 3). In an *in vitro,* two-taxa model system of small intestinal bacterial overgrowth, they showed how a “dietary” perturbation, namely, increasing media glucose levels, allowed *Klebsiella pneumoniae* (*K. pneumoniae*) to perform aerobic glucose metabolism, completely consuming available oxygen in the media. The consequently low-oxygen state permitted the growth of *Bacteroides thetaiotaomicron* (*B. thetaiotaomicron*), an obligate anaerobe, which then broke down dextran in the media into additional glucose. Even after exogenous media glucose levels were reduced to their initial state, the system did not return to baseline because sufficient glucose was produced by *B. thetaiotaomicron* through dextran breakdown, and *K. pneumoniae* could continue to consume all the oxygen. Thus, the system could not reverse course by stopping the perturbation; in order to return to baseline, oxygen levels had to be increased.

This dependence of the current state on not only the current environmental conditions, but also the previous state, is known as “hysteresis.” Although parsing the mechanisms that drive hysteresis is far more complex in natural gut ecosystems with hundreds of taxa, one study from Thaiss et al. identified a hysteretic microbiome signature that persists after dieting and weight loss in formerly obese mice.^149^ Reversing weight gain and normalizing body fat, cholesterol, glucose tolerance, and insulin levels are insufficient to bring the microbiota back to its initial state. Instead, using a metabolomics approach, the authors identified a persistent deficiency of two dietary flavonoids in dysbiotic mice, which, when supplemented, restored the taxonomic and functional properties of the microbiota. Although additional studies are needed to extend these findings to humans, these results suggest that, with a better understanding of how a given perturbation affects ecosystem conditions, we may be able to identify orthogonal pathways of intervention to promote recovery in hysteretic systems, especially when brute-force efforts to reverse perturbation have been ineffective.

## Where: what are the salient features of the ecosystem where the perturbation will take place?

**Figure 4.**
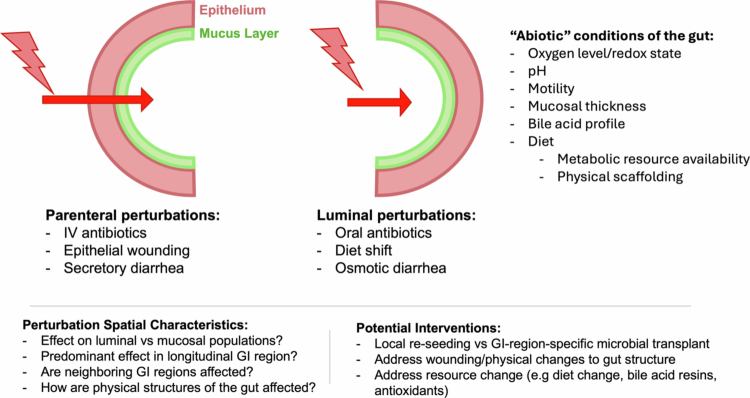
**GI disturbance can be spatially heterogeneous.** As depicted in this schematic cross-section of the intestine, perturbations to the GI tract may differentially affect luminal and microbial communities, depending on the specific nature of the perturbation. Perturbations such as IV antibiotics and epithelial wounding affect the GI tract primarily via the epithelial surface, as factors such as antibiotics, immune effectors, and oxygen diffuse through the epithelium and mucosal layer prior to entering the luminal space. Other perturbations, such as poorly absorbed oral antibiotics or dietary shifts, primarily impact the luminal contents, changing the availability of metabolic resources and physical scaffolding upon which microbial taxa can adhere. Other perturbations may differentially affect proximal or distal regions of the GI tract. Surgery, for instance, exerts a locally specific impact on the GI ecosystem, bringing distant microbial populations and host physiological functions into new juxtaposition. Interventions that explicitly consider GI ecosystem conditions and regionality might include region-specific microbial transplant (e.g., jejunal vs. cecal vs. fecal microbial transplant) or interventions that seek to directly ameliorate changes to ecosystem conditions, such as hydrogels designed to protect wounded epithelial surfaces, dietary interventions designed to provide resources and scaffolding for microbial taxa, or antioxidant administration that can restore the redox environment and anaerobic state of the gut.

Another essential consideration in the ecological process of microbiome recovery is where: what is the “abiotic,” or non-microbial, environment, such as in the specific location where the perturbation will take place? How will this environment change when perturbed? Even within the gut, the local environment must be carefully characterized across different regions and microhabitats of the GI tract. Gradients in oxygen levels^150^^,^^151^, pH^152^, mucus thickness^153^, motility^154^, bile acid production, and absorption^155^, and antimicrobial peptide production^156^, and more have been well documented along the proximal-distal axis of the GI tract, with corresponding differences in the microbial inhabitants of each region^157^. The mediolateral axis of the GI tract likewise maintains important physiologic gradients, such as diffusion of oxygen^158^, secretion of antimicrobial peptides, IgA, and other immune effectors^156^, and the presence of mucus or dietary components that provide physical surfaces for bacteria to adhere to^159^, with distinct populations of bacterial^113^^,^^160^, fungal^161^, and viral^162^^,^^163^ taxa across mucosal and luminal niches (Figure 4).

All of these factors, which are fundamental reflections of host physiology, are at least partially constrained by host genetics. For instance, the gene *CARD15*, closely associated with the risk for Crohn's disease, regulates NOD2 antimicrobial immune signaling and barrier function in the ileum and colon.^164^ Genes encoding bile acid transporters such as *Slc10a2* or the broader bile acid transcriptional regulator *FXR* modulate the bile acid profile, with associated impacts on microbiome composition.^165^^,^^166^ Although the evidence in humans is conflicting, some researchers argue that *FUT2*, a gene that determines whether blood-type antigens are secreted into the gut or not, mediates microbiota composition via bacterial adhesion to and metabolism of secreted antigen.^167^^,^^168^ Individuals with mutations in genes underlying these environmental features of the gut may have altered physiology that predisposes to dysbiosis and that must be addressed in order to facilitate recovery.

To demonstrate the significance of spatial effects on microbiome recovery, we may look again to an example using antibiotic treatment. In examples from both humans and mice, broad-spectrum oral antibiotic regimens induce region-specific changes in the microbiota, both longitudinally^52^ and mediolaterally^169^. Orally delivered antibiotics that are poorly absorbed in the GI tract, such as aminoglycosides, are often used to clear the gut prior to surgery or to prevent opportunistic infection in critically ill patients in intensive care units^170^^,^^171^. One study that examined the collateral damage of the aminoglycoside neomycin in combination with bacitracin on the microbiota in mice found that these antibiotics more severely diminish and alter the luminal microbiota compared to mucosal populations^172^. Another study in humans using the aminoglycoside paromomycin similarly identified differential effects on the microbiota across the luminal and mucosal compartments^173^. A comparison of oral and intravenous amoxicillin and levofloxacin in mice found that although mice underwent similar changes to microbiome structure regardless of the route of administration, the time to recovery was reduced for mice receiving intravenous antibiotics.^174^ Thus, microbiome robustness to antibiotic perturbation and recovery dynamics afterward may depend on the local bioavailability and clearance of that antibiotic across GI regions.

Dietary shifts can also induce “abiotic” changes within the gut. Fiber is a well-studied example that shapes the “abiotic” environment of the distal GI tract in a multitude of predictable ways. It provides physical scaffolding upon which bacteria can grow^175^. Its many reducing sugar monomers can act as electron donors, decreasing the colonic redox potential^176^^,^^177^, as well as providing additional carbohydrate metabolism niches for diverse bacterial taxa^178-181^. Short-chain fatty acid (SCFA) byproducts of fiber like butyrate can prevent the pro-inflammatory differentiation of human monocytes into dendritic cells and inhibit T cell proliferation^182^, while their oxidation by colonocytes maintains a healthy and anaerobic colon^127^^,^^183^. The absence of fiber can promote the digestion of the protective epithelial mucus layer by microbial taxa that otherwise localize more luminally, rendering the epithelium more vulnerable to infiltration by microbes and consequent inflammation^131^. Mouse and human studies suggest that reintroducing fiber after antibiotic perturbation plays an essential role in promoting microbiome recovery by restoring the redox state of the gut and producing metabolic byproducts that facilitate the microbial successional process^101^^,^^176^^,^^177^^,^^184^. These impacts have been recapitulated across a variety of fiber sources, including whole oats^185^ and diverse unprocessed plant fiber^101^, pectin^177^, inulin^186^, and a cocktail of isolated plant fibers, including cellulose, levan, dextrin, pectin, inulin, beta-glucan, and arabinoxylan^176^. Further research is needed to compare the effects of different fiber sources more explicitly. Recent work further suggests that, at least for a relatively limited course of antibiotic treatment, dietary intervention need not be prolonged—a short-term intervention on the scale of days to weeks may be sufficient to stimulate recovery.^185^^,^^187^

Dietary fat has been shown to increase oxidative stress in the gut^188^, as well as to promote host secretion of primary bile acids, with increased conversion into secondary bile acids by the colonic microbiota^189^. As many bile acids are directly bactericidal to certain taxa, a shifted bile acid profile can dramatically impact microbiome community assembly dynamics^190^^,^^191^. The administration of exogenous bile acids, such as obeticholic acid, has recently been explored in clinical trials as a method of manipulating microbiome composition.^192^ Buffie et al. showed that restoring a *Clostridial* taxon capable of 7⍺-dehydroxylating bile acids was protective against *C. difficile* infection in mice with antibiotic-induced dysbiosis.^193^

Immune system activity is a variable feature of the GI habitat that plays an important role in mediating microbiome homeostasis through both innate and adaptive mechanisms^194^. Continuously bombarded with foreign antigens from digested material, the GI tract maintains a physiologic baseline level of low-grade immune activation^195^. Perturbations that impact the delicately balanced immune environment of the gut may have consequences for microbiome restoration. For instance, antibiotic perturbation of the microbiota is associated with a corresponding reduction in baseline intestinal immune activation, predisposing hosts to opportunistic infection that can further inhibit spontaneous recovery of the native microbiota^196^. Systemic chemotherapy, which severely diminishes the immune system, is also associated with severe gut microbiome dysbiosis.^197^

Inflammation of the GI tract induces a broad array of environmental changes in the gut environment, many of which ultimately derive from diffuse damage to the mucosa and wounding of epithelial surfaces. The local environment becomes more aerobic because of increased epithelial blood flow and the diffusion of oxygen across a damaged mucosal barrier^198^. By targeting these environmental changes, we might identify new strategies to promote microbiome recovery. For instance, promising preliminary work from Sun et al. explored the potential of exogenous hydrogels that can coat and protect epithelial surfaces, much in the same way that a healthy colonic mucus layer does^199^. This could act as a barrier to microbial infiltration of tissues and the associated pro-inflammatory response while also minimizing the diffusion of oxygen into the lumen, allowing for the restoration of a healthy, anaerobic colonic community. Other recent work has examined the possibility of administering mesenchymal stem cells^200-203^, application of intestinal organoid cultures^204^^,^^205^, or intestinal growth factors such as teduglutide^206^^,^^207^ to promote epithelial wound healing during inflammation.^208^ One group introduced a novel antioxidant that specifically targets mitochondrial-produced reactive oxygen species, with improvement of epithelial barrier function in an e*x vitro* experimental model using colonic biopsies.^209^

Commonly used medications such as proton pump inhibitors (PPIs), which increase intestinal pH, bile acid sequestrants, and osmotic laxatives, provide a clinical example of the impact of abiotic interventions on the microbiota. PPIs exert a well-characterized impact on the human gut microbiota, with increases in an array of facultative anaerobes, such as *Staphylococcus*, *Streptococcus*, and *Escherischia.*^210^ Although no studies have explicitly evaluated whether PPI-induced dysbiosis impairs recovery of the microbiota after antibiotic treatment, one study determined that the interaction of PPIs and antibiotics synergistically increases patients’ risk of opportunistic *C. difficile* infection, which strongly suggests underlying microbiome dysbiosis^211^. In patients with primary biliary cholangitis, a positive clinical response to bile acid sequestrants appeared to be dependent on specific changes to the gut microbiota related to the modified bile acid profile.^212^ Osmotic laxatives pull water into the gut lumen owing to the high osmotic pressure of poorly absorbed solutes and are commonly used to treat constipation or for bowel preparation prior to surgery or endoscopy, often in conjunction with antibiotics. In mice, osmotic laxatives have been shown to flush microbial taxa such as the abundant *Muribaculaceae (S24-7)* family to the point of extinction from the GI tract, causing persistent dysbiosis that is irreversible without intervention,^137^ and can increase colonization of *C. difficile*^213^. In humans, osmotic laxatives affect microbiome structure and function, but this effect appears to be reversible.^35^^,^^136^ Additional studies investigating whether or how microbiota dysbiosis resulting from bowel preparation might affect resilience to antibiotic treatment or surgical insult to the GI tract are needed.

There are many opportunities to investigate how the local GI microenvironment affects microbiome restoration after surgical perturbation. Surgeries such as ileocecectomies, in which the small and large bowels are anastomosed, create unique new juxtapositions of host and microbial physiology: the more absorptive and metabolically active small bowel tissue becomes immediately adjacent to large bowel tissue with more secretory and immunologically active tissues. Little work has yet been done to characterize how the admixture of these two microbial communities ultimately resolves, although recent work from DeLeon et al. indicates that the transplantation of region-specific microbiota can actively reprogram epithelial cellular identity and function^81^, which could in turn impact microbial assembly dynamics over time. To acquire human small intestinal material without relying on invasive upper endoscopy, ingestible pill-shaped sampling devices such as CapScan^214^ or the SIMBA capsule,^215^ both recently developed for longitudinal sampling of the GI tract, could plausibly be adapted for the collection of region-specific contents. Delivery of transplanted material to the upper GI tract could similarly rely on pH-sensitive capsule coatings.

Proctocolectomy with ileal pouch anal anastomosis (IPAA) surgery in patients with UC provides another example of an “abiotic” change resulting from surgical intervention: after complete resection of the colon and rectum and functionalization of an ileal J-pouch to restore fecal continence, the ileal pouch begins to take on a cellular and functional role more similar to that of the colon, with slower motility, reduced oxygen levels, and increased expression of extracellular matrix remodeling and pro-inflammatory pathways^216^. Microbial communities, in turn, respond to these changes, becoming more colonic, and in some cases transitioning to dysbiosis that provokes UC-like inflammation of the ileal pouch.^217-219^ By modulating the local environment of the terminal ileal pouch to control “colon-ization,” perhaps we might be able to reduce the risk of pouchitis.

## When: what is the time course of perturbation and recovery?

**Figure 5.**
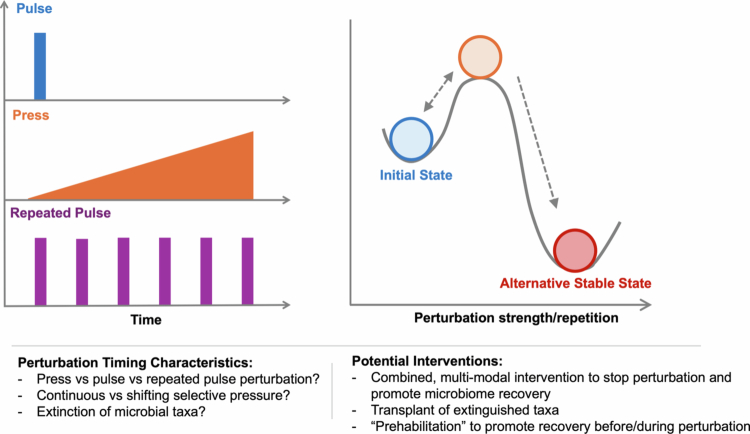
**The duration and frequency of perturbation affect the severity of impact and recovery dynamics.** Perturbations may be characterized as a single pulse (brief, finite perturbation), a press (prolonged shift in conditions), or a repeated pulse (multiple finite perturbations that may or may not allow sufficient time for complete recovery between intervals). The magnitude, duration, and frequency of perturbation may affect whether a community exhibits resilience, returning to its initial state, or whether it transitions to an alternative stable state, from which return to the initial state would require an intervention of far greater magnitude. Effective interventions will account for the time course of perturbation, potentially including measures to halt the perturbation itself in order to kickstart recovery or “prehabilitation” strategies before or during repeated perturbation in order to continuously facilitate recovery.

Ecologists categorize most ecosystem disturbances as “pulse” or “press” perturbations^220^ (Figure 5). A pulse perturbation represents a discrete, finite, and relatively brief disturbance. A repeated pulse reflects multiple sequential disturbances that may or may not allow for complete interim recovery^221^, and a press perturbation represents a prolonged disturbance that may extend arbitrarily into the future or even indefinitely. Most obviously, the varying durations of pulse or press perturbations affect the severity of the perturbation’s impact on the gut ecosystem—both in terms of the “biotic” component, which we use to refer to the abundances of microbial taxa, as well as the non-microbial, “abiotic” conditions of the GI habitat. While many relatively stable communities may rebound quickly after a brief pulse perturbation^63^^,^^121^^,^^222^^,^^223^, a press may exert severe enough pressure to tip the system towards an alternative state, which may or may not be stable itself.^224-226^

For example, while a brief pulse of antibiotics promotes the expansion of antibiotic-resistant organisms and the horizontal transfer of ARGs, without continued selective pressure from persistent administration of antibiotics, the community taxonomic structure often returns to near-baseline, although ARGs typically persist^121^^,^^227^. However, prolonged antibiotic treatment can drive the extinction of bacterial taxa from the community, leading to persistent changes in the microbial ecosystem structure.^62^^,^^224^^,^^228^^,^^229^ Similarly, a short-term pulse of a high-fat, low-fiber diet causes reversible changes to both the resource environment of the gut and the microbial community, including a transient increase in *Firmicutes* and loss of *Bacteroidetes* taxa^222^. By contrast, a sustained press of high-fat, low-fiber diet across generations of mice induces the extinction of taxa that are not reversible without intervention.^61^

The time course of recovery, by which we mean when recovery can initiate and how it progresses over time, has been insufficiently investigated for most types of disturbances. Must a perturbation have ceased for recovery to begin, or, particularly in cases of sustained press perturbations, and particularly when microbial dysbiosis itself feeds back and contributes to the perturbation, might we be able to intervene to kickstart recovery from the dysbiotic state? Inflammation in ulcerative colitis (UC), for example, both causes microbial dysbiosis and is worsened by it^59^^,^^230-232^. The standard of care for UC is to address inflammation, typically via immunosuppressive agents, without any intervention to address the dysbiotic microbiota^147^. These measures are effective for some individuals, but many individuals either respond poorly to treatment from the time of initiation or lose response over time^233^. Perhaps a multimodal treatment strategy that coordinates immunosuppressive interventions with measures to promote regrowth to a eubiotic state (e.g., dietary intervention, microbial replacement) could more effectively kick start the recovery phase. Although few studies have explicitly explored the impact of multimodal therapy versus immunomodulatory therapy alone, retrospective clinical studies suggest that the microbiome state may predict the efficacy of anti-TNF agents in inflammatory bowel diseases^234-236^ and spondyloarthritis.^237^

For discrete pulse perturbations such as a short course of antibiotics or bowel washout, it may be reasonable to postpone interventions directed towards the microbiota until after the perturbation. However, there is increasing evidence, particularly in elective peri-operative settings, supporting the benefits of “prehabilitation” or “peri-habilitation” interventions, in which the microbiota and/or GI tract are proactively conditioned prior to and possibly extending through and beyond the perturbation. For example, a mouse study from Keskey et al. showed that a 7-d dietary prehabilitation protocol in which dietary fiber and fat levels were optimized led to rapid changes in microbiota composition with recovery of butyrate-producing taxa and improved survival after partial hepatectomy^238^. Another study showed that 6 weeks of dietary prehabilitation in mice prior to colonic surgery and anastomosis reduced rates of anastomotic leak compared to mice without dietary prehabilitation^239^, with subsequent mechanistic work identifying collagenolytic bacteria as likely contributors to this effect^240^. A retrospective study in humans recapitulated the protective effect of fiber supplementation against surgical complications^241^. Markers of microbiome readiness for surgery could therefore include factors such as the abundance or diversity of collagenolytic bacteria or genes, metabolic readouts such as butyrate production,^238^ or other markers that can be validated in human cohorts. Ongoing clinical trials are actively investigating whether or how diet can be used to prehabilitate the microbiota of patients undergoing major colorectal or other abdominal surgeries (NCT06349590 and NCT05027763 at clinicaltrials.gov).

Finally, a severely understudied feature of microbiome recovery is the progression of recovery over time. Owing largely to limitations in the cost of granular time-course sampling, much existing research on microbiota recovery evaluates only a single endpoint, at which time the microbiota is determined to have recovered or not, painting a binary picture of the recovery process. We argue that the transitional stages between dysbiosis and eubiosis hold tremendous insight into the ecology of recovery and the optimal timing and structure of interventions to promote recovery. By performing repeat time course sampling, at least in a research context if not yet in clinical settings, we can characterize the order and progression of both the “biotic” (microbial taxa) and “abiotic” (ecosystem conditions such as resource availability, oxygen levels, pH, motility, and host immmunity) components of the ecosystem and determine how generalizable these patterns are across individuals undergoing the same perturbation. This may allow us to identify taxonomic or metabolic milestones that must be reached before the community can progress to the next stages, which we refer to as a “successional” model of recovery.

Successional theory has primarily been applied to the microbiome in the context of infant gut development, in which an orderly sequence of microbial taxa colonize and establish in the gut, gradually evolving over time with factors such as immune development and the introduction of solid foods^242^^,^^243^. The infant gut constitutes a case of “primary succession”, or the initial colonization and assembly of a community from a fully naïve state^244^. In contrast, microbiome recovery after perturbation falls under the umbrella of “secondary succession” or the reestablishment of a previously stable community after disturbance. In the context of secondary succession, there may be more condition-specific barriers to recovery, such as persistently modified abiotic conditions, or priority effects in which the remaining taxa experience a relative advantage.^245^

Nevertheless, with the caveat that functional redundancy may undermine the identification of rigidly sequential microbial recolonization, limited but emerging evidence supports the possibility of function-driven microbial succession in adults. For instance, two studies of microbiome recovery after antibiotics in mice^101^ and in humans^246^ indicated that early utilization of dietary fiber and complex carbohydrates may facilitate a metabolically-driven cascade of community cross-feeding and staged diversification. One study of FMT in mice demonstrated orderly succession of early and late colonizers,^247^ and another FMT study in humans with recurrent *C. difficile* infection indicated that, in patients for whom FMT successfully changed microbiome structure, the community underwent a replicable succession of microbiome functions, beginning with colonization of “nexus” species that engineer the ecosystem to allow for the successive proliferation of bile acid metabolizers, sporulators, and short-chain fatty acid producers.^79^ This study also showed that the presence of a fungal taxon, *Yarrowia*, could inhibit the progression of the successional process.

## Conceptual advances and future directions

Existing conceptual frameworks for understanding microbiome stability and resilience to perturbation rely heavily on the premise of stability landscapes (depicted in Figure 5), initially proposed in macroecology by Holling^248^, and adapted to describe gut microbial ecosystems^249^. Stability landscape models represent ecosystem state as a ball on a surface with hills and valleys. Disturbance acts to push the ball around this landscape, which can climb hills or settle in valleys depending on the magnitude and duration of the perturbation. A stable equilibrium returns to its initial state after perturbation, and an unstable equilibrium transitions to an alternative state. This framework has been primarily applied as a conceptual tool, allowing researchers to identify ecosystem features that are associated with communities that were experimentally determined to be stable or unstable after perturbation^250^^,^^251^. A small number of studies have performed computational implementations of stability landscapes using gut microbiome data, but these are largely phenomenological approaches in which large datasets are used to tally taxonomic transitions between states and predict the likelihood of transition from one to another, with minimal mechanistic insight into why a given state may be stable or unstable.^225^^,^^226^^,^^252^

A major shortcoming in the way that stability landscape theory has been utilized to date in the microbiome sphere is that it tends to center inquiry on features associated with equilibria (i.e., initial or end states) and neglects the dynamic process of transition between states. By incorporating the *who, what, where,* and *when* heuristic into a stable landscape framework, we are forced to confront the dynamic ecology of transitions and can identify more complex trajectories between stable states. For example, although the schematic stability landscape presented in Figure 5 presents a single axis of ecosystem variation and it becomes difficult to visualize more than 2 axes simultaneously, in theory, this model can accommodate as many ecosystem features as are available in a dataset. What appears to be an uphill climb in one dimension may simultaneously entail a downhill stroll in another, indicating the presence of trade-offs whose net impact dictates the hills and valleys of the stability landscape. The primary factors driving the transition from state to state could even vary over the course of the transition. By incorporating more information about microbial agents and functions, disturbance characteristics, and abiotic conditions, particularly across intermediate stages of disturbance and recovery, we can see how each of these factors interacts with and constrains one another.

Computationally, the modeling approaches presented above (for instance, from Shaw et al. ^225^) could be adapted to incorporate high-dimensional multi-omics data on a more diverse array of ecosystem conditions, such as taxonomic and gene profiles, metabolomics and proteomics data, other immune profiling, or measurements of abiotic gut factors. This could then be used to identify the strongest attractors across a multi-dimensional stability landscape, which we might think of as potential targets for staged intervention strategies. Alternatively, machine-learning approaches such as random forest classifiers with feature selection could effectively accommodate a similar dataset^253^. Such approaches could perhaps even integrate and synthesize information gathered from across published experiments to more effectively mine information.^254^

Truly personalized interventions will require the ability to easily and regularly monitor microbiota health. Although fecal DNA sequencing is increasingly affordable, taxonomic information about microbiome composition is incomplete without a functional measure of microbiome metabolic output^55^. Point-of-care tools to evaluate microbiome functional output, perhaps via targeted metabolomics^255^, must be developed to fill this gap. To acquire more granular data on abiotic and regional conditions of the gut may similarly require the development and broader application of tools such as CapScan for longitudinal GI sampling^214^. Tools are currently in development to infer dietary habits from residual food-derived DNA in stool, which could aid tremendously in characterizing dietary input in a high-throughput manner.^256^ With such tools, granular time course information on recovery can be generated to evaluate the progress of microbiome recovery and course-correct as necessary in real time.

## Conclusions

The microbiota holds immense promise as a modifiable driver of human health, but we have yet to develop effective and reliable means of microbiome modification. The studies and ideas presented in this Review underscore the challenges and opportunities that arise when all elements of disturbance and recovery are appropriately considered. By framing recovery as a dynamic ecological process, we hope to focus future efforts on the development of multimodal strategies to guide the regrowth of microbial communities over time rather than seeking an apocryphal panacea to reverse dysbiosis in one step. As we continue to expand our understanding of all the elements of this dynamic process, we may hope to one day comprehend and manage the microbiota as a routine element of standard medical practice.
